# Autophagosome Proteins LC3A, LC3B and LC3C Have Distinct Subcellular Distribution Kinetics and Expression in Cancer Cell Lines

**DOI:** 10.1371/journal.pone.0137675

**Published:** 2015-09-17

**Authors:** Michael I. Koukourakis, Dimitra Kalamida, Alexandra Giatromanolaki, Christos E. Zois, Efthimios Sivridis, Stamatia Pouliliou, Achilleas Mitrakas, Kevin C. Gatter, Adrian L. Harris

**Affiliations:** 1 Department of Radiotherapy-Oncology, Democritus University of Thrace Medical School, University General Hospital of Alexandroupolis, Alexandroupolis, Greece; 2 Department of Pathology, Democritus University of Thrace Medical School, University General Hospital of Alexandroupolis, Alexandroupolis, Greece; 3 Nuffield Department of Cellular Sciences, John Raddcliffe Hospital, Headington, Oxford, OX3 9DU, United Kingdom; 4 Cancer Research United Kingdom Molecular Oncology Laboratories, Weatherall Institute of Molecular Medicine, University of Oxford, Headington, Oxford, OX3 9DU, United Kingdom; IISER-TVM, INDIA

## Abstract

LC3s (MAP1-LC3A, B and C) are structural proteins of autophagosomal membranes, widely used as biomarkers of autophagy. Whether these three LC3 proteins have a similar biological role in autophagy remains obscure. We examine in parallel the subcellular expression patterns of the three LC3 proteins in a panel of human cancer cell lines, as well as in normal MRC5 fibroblasts and HUVEC, using confocal microscopy and western blot analysis of cell fractions. In the cytoplasm, there was a minimal co-localization between LC3A, B and C staining, suggesting that the relevant autophagosomes are formed by only one out of the three LC3 proteins. LC3A showed a perinuclear and nuclear localization, while LC3B was equally distributed throughout the cytoplasm and localized in the nucleolar regions. LC3C was located in the cytoplasm and strongly in the nuclei (excluding nucleoli), where it extensively co-localized with the LC3A and the Beclin-1 autophagy initiating protein. Beclin 1 is known to contain a nuclear trafficking signal. Blocking nuclear export function by Leptomycin B resulted in nuclear accumulation of all LC3 and Beclin-1 proteins, while Ivermectin that blocks nuclear import showed reduction of accumulation, but not in all cell lines. Since endogenous LC3 proteins are used as major markers of autophagy in clinical studies and cell lines, it is essential to check the specificity of the antibodies used, as the kinetics of these molecules are not identical and may have distinct biological roles. The distinct subcellular expression patterns of LC3s provide a basis for further studies.

## Introduction

Autophagy is a major intracellular pathway for the degradation and recycling of long-lived proteins and entire organelles [1,2]. LC3s (MAP1-LC3s) are structural proteins of autophagosomal membranes. The human LC3 gene family has three members, LC3A, LC3B and LC3C, whilst two variants of the LC3A protein have been identified [3,4]. The human LC3 gene family has three members the LC3A, LC3B and LC3C while in a recent paper has been reported five members, LC3A (variant-1, variant-2), LC3B, LC3B2 and LC3C [4]. The form LC3-II is one of the main components of the autophagosome membrane (LC3A-II and LC3B-II, also LC3C-II but not studied here) that resides in both the inner and outer site of the membrane. The LC3-II is derived from a proLC3 ~30KDa protein after cleavage by autophagin Atg4 to produce the active cytosolic form LC3-I. This in its turn is activated by Atg7, and then transferred to Atg3, a second E2-like enzyme, becoming a membrane-bound form, LC3-II [5]. After autophagosome formation, the LC3-II located in the outer site is released to the cytosol and the LC3-II located in the inner site is degraded by hydrolases [6]. In this latter form, LC3-II localizes on the spherical autophagosomal and autolysosomal membranes, forming a suitable marker of autophagic activity [6,7].

Whether these three LC3 proteins have a similar biological role in autophagy or other pathways remains obscure. In the literature, most studies focus on an overall LC3 expression, without reporting on the specificity of antibodies used, based on the arbitrary assumption that A and B forms are equivalent. In the current study we examine, after extensively validating antibody specificity, the expression in parallel of the three LC3A, B and C proteins in human cancer cell lines, showing distinct patterns of sub-cellular localization, suggesting a distinct biological role of these sister proteins.

## Results

### Identification of LC3A and B specific antibodies

**Fig 1:**
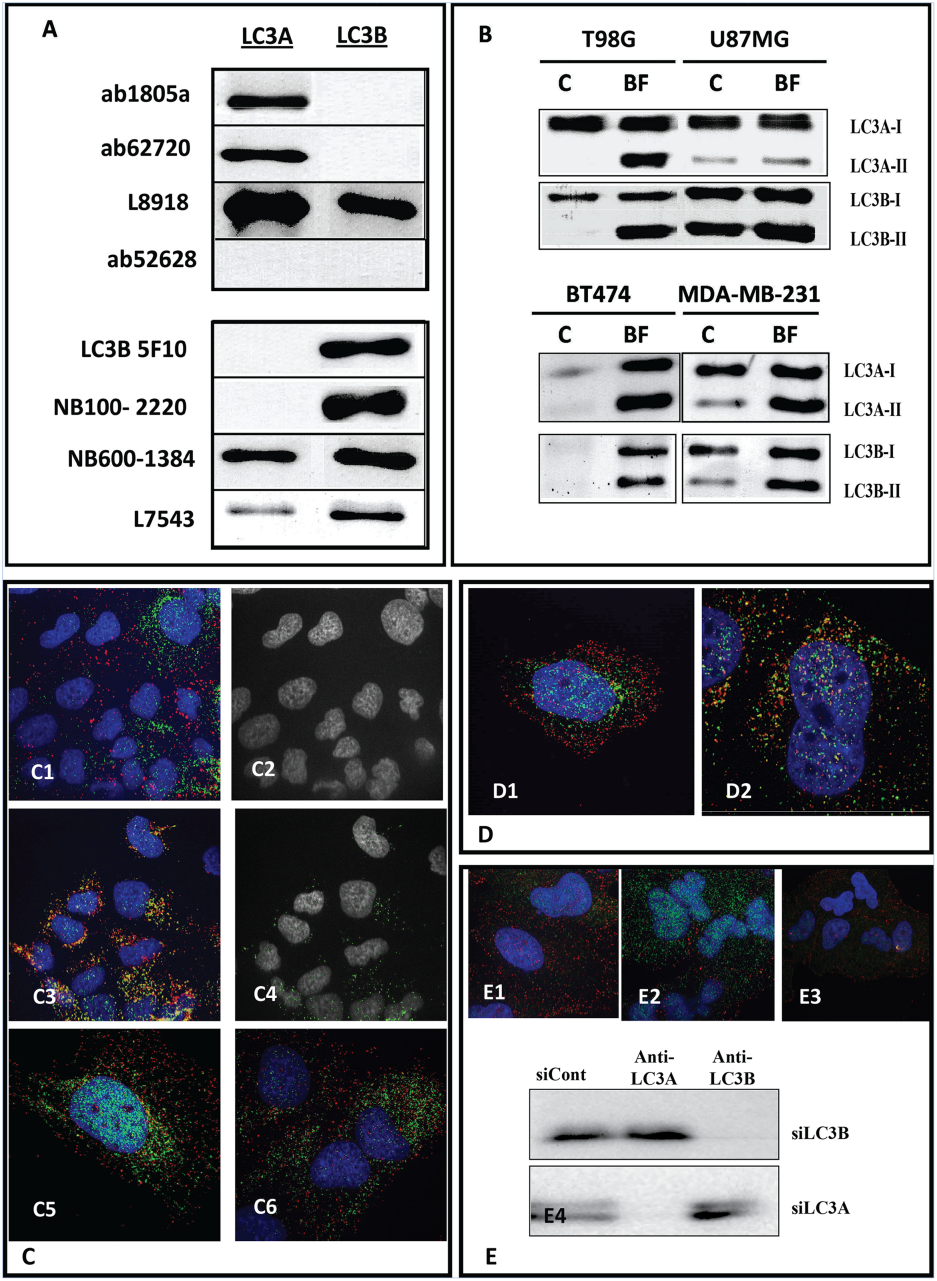
**(A)** Specificity of various anti-LC3 antibodies tested against recombinant LC3A and LC3B proteins. **(B)** The LC3A and LC3B processing after 24h of Bafilomycin (100nM) treatment in glioma and breast cancer cell lines, using the LC3A specific ab62720 and of the LC3B specific 5F10 antibodies **(C)** Double immunofluorecence in the A549 cell line using the ab62720 recognizing exclusively the LC3A protein and the 5F10 antibody that reacts exclusively with LC3B. Noted a clearly distinct expression of LC3A in green vacuoles with perinuclear/nuclear localization and of LC3B red vacuoles that have a diffuse, throughout the cytoplasm, localization. There are no autophagosomes composed of both LC3A and LC3B proteins (c1,c2), whilst LC3A and LAMP2a show extensive colocalization (c3,c4). The distinct identity of LC3A and LC3B autophagosomes was also confirmed under acidic conditions (c5) and after exposure to bafilomycin (c6). **(D)** Double immunofluorecence in A549 cell line using the ab62720 recognizing exclusively the LC3A protein and the NB600-1384 that reacts with both LC3A and LC3B proteins. Noted that the majority of the perinuclear/nuclear LC3A (green) vacuoles show double immunofluorescence (yellow), a result of double LC3A/LC3B reactivity that produces the NB600-1384 antibody. **(E)** LC3A and LC3B siRNAs specifically block the expression of the LC3A and LC3B proteins, respectively, in A549 cell line (E1,2,3). In E4 the reactivity of LC3A (ab62720 Ab) and of the LC3B (5F10 antibody) is shown, following silencing of the LC3A or of the LC3B genes, in the A549 cell line.

The specificity of the antibodies tested against to the commercial available human recombinant proteins LC3A and LC3B is shown in Fig 1A. The anti-LC3A antibodies (ap1805a and ab62720) are specific to the protein LC3A and the anti-LC3B antibodies (5F10 and NB100-2220) are specific to the protein LC3B. The L8918, NB600-1384 and L7543 can bind either to the LC3A or LC3B, but with different sensitivity, while the ab52628 failed to detect either of the two isoforms under the same conditions.

We focused on both LC3A and LC3B proteins to analyze autophagic response to Bafilomycin in cell lines using the two antibodies that selectively recognize these isoforms. The two isoforms showed different base line and response profile expression (Fig 1B) Under bafilomycin stress there was accumulation of the LC3A-II and LC3B-II in all cell lines, suggesting that both proteins could be used to assess autophagic flux (Fig 1B). The T98G and BT474 cell lines have higher autophagic flux (as assessed either by LC3A or LC3B immunoblotting) compared with the U87MG and MDA-MB-231 respectively (Fig 1B).

### LC3A and LC3B expressing autophagosomes

Confocal microscopy with double LC3A/B immunofluorescence, using LC3-type specific antibodies, revealed that LC3A and LC3B autophagosomes are distinct and that there are no autophagosomes expressing both proteins (Fig 1C1 and 1C2). This finding was universal in all cell lines examined. Colocalization analysis revealed a percentage <1% whilst LC3A/LAMP2A colocalization in control cells was higher than 20% (Fig 1C3 and 1C4).

The distinct identity of LC3A versus LC3B autophagosomes was also confirmed after intensification of autophagy under acidic conditions (Fig 1C5) and after autophagy flux blocking following exposure to Bafilomycin (Fig 1C6). In contrast, non-specific antibodies showed a wide superimposing expression, a result of dual recognition of LC3A and LC3B by the antibody (Fig 1D1 and 1D2).

Using siRNA for LC3A and LC3B, these specifically blocked the accumulation of LC3A or of LC3B autophagosomes, respectively (Fig 1E1, 1E2 and 1E3). Silencing of LC3A or of LC3B confirmed loss of the identification of the respective proteins in western blots (Fig 1E4).

### Cytoplasmic LC3A and LC3B localization patterns

**Fig 2:**
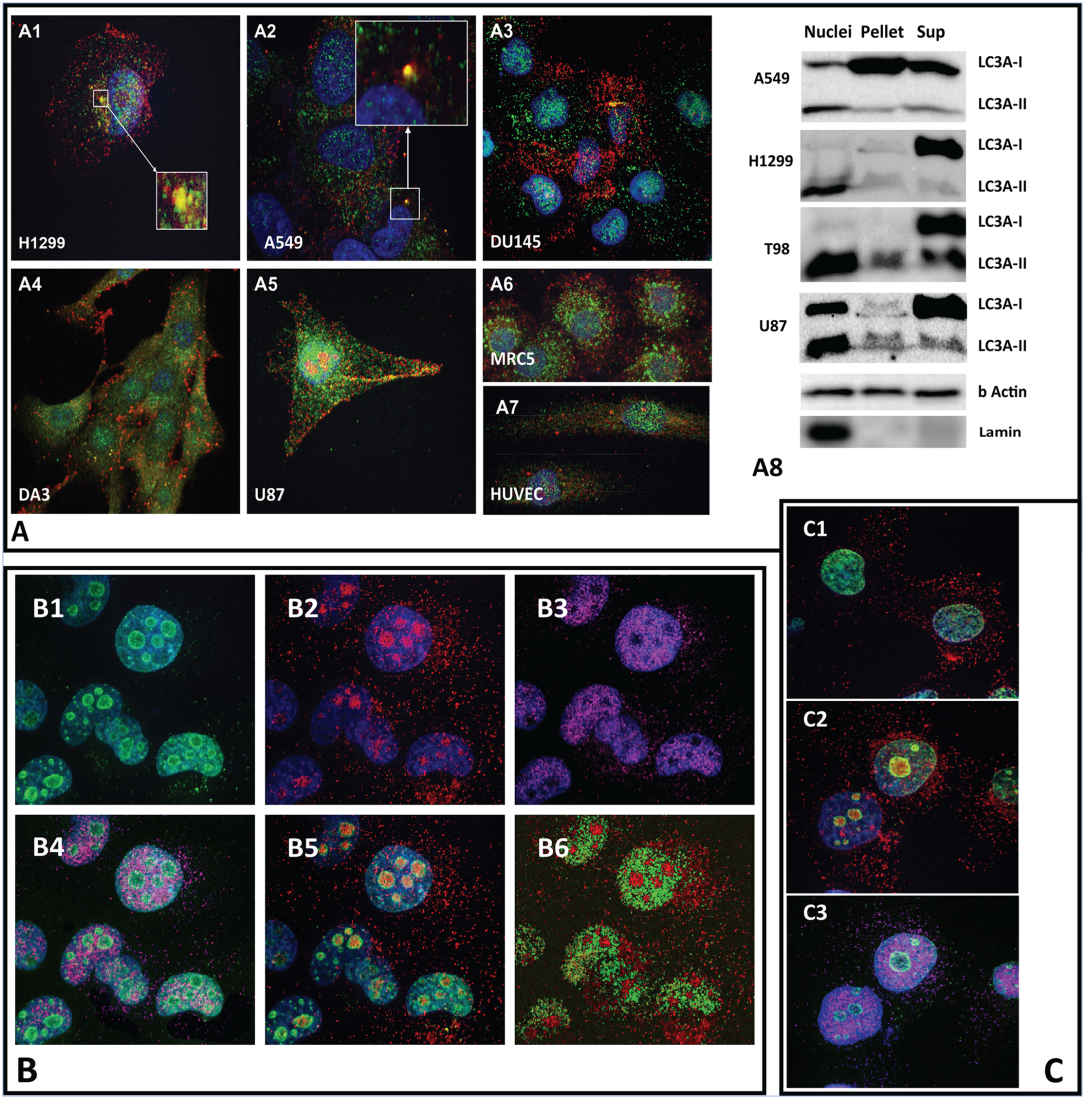
**(A)** Confocal double immunofluorescence for LC3A (green) and LC3B (red) in various cell lines. Noted the LC3A accumulation in the perinuclear area, while LC3B is distributed throughout the cytoplasm (A1-7). LC3 aggregates, suggestive of small LC3A+ autophagosomes included in LC3B+ autophagosomes, are occasionally present (boxes in A1,2). Western blots confirm the nuclear presence of LC3A in the nuclei, mainly with the LC3A-II form (A8). **(B)** Nucleoli are stained with the MIB1/green (B1), the LC3B/red (B2), but not the LC3A/violet antibody (B3); LC3A specific ap1805a and of the LC3B specific 5F10 antibodies were used. This is confirmed in double immunostaining for LC3A/MIB1 (B4), LC3B/MIB1 (B5) and LC3A/LC3B (B6). Noted that LC3B stains the internal nucleolar areas, while MIB1 the peripheral. **(C)** Actinomycin D damages nucleoli and disrupts the nucleaolar LC3B and MIB1 localization (C1). Acidic conditions (pH = 6.5; C2) or hyperthermia (40°C; C3) do not abrogate the distribution of LC3B in the nucleoli nor of LC3A in the nuclei. Lamin is also used as a control to confirm the presence of nuclei only in the nuclear fraction.

The distribution of LC3A and LC3B in the cytoplasm was quite distinct. LC3A was mainly accumulated in the perinuclear area, while LC3B was evenly distributed throughout the cytoplasm. This phenomenon was evident in all cell lines examined and, as shown by cytoplasmic expression intensity analysis, perinuclear LC3A reached up to 90% (range 60–90%) of the total protein cytoplasmic content (Fig 2A1–2A7). Occasionally, small LC3A+ autophagosomes included in LC3B+ autophagosomes were evident in the cytoplasm (Fig 2A1 and 2A2).

### Nuclear LC3A and LC3B localization patterns

LC3A was clearly localized in the nuclei of all cancer cell lines examined, including the HUVEC s and the human MRC5 fibroblast line (Fig 2A1–2A7). Western blot analysis after triple fractionation (nuclear—pellet—supernatant) showed clearly presence of the LC3A proteins in the nuclei, more evident as LC3A-II form, that was more prominent in the glioblastoma cells (Fig 2A8).

**Fig 3:**
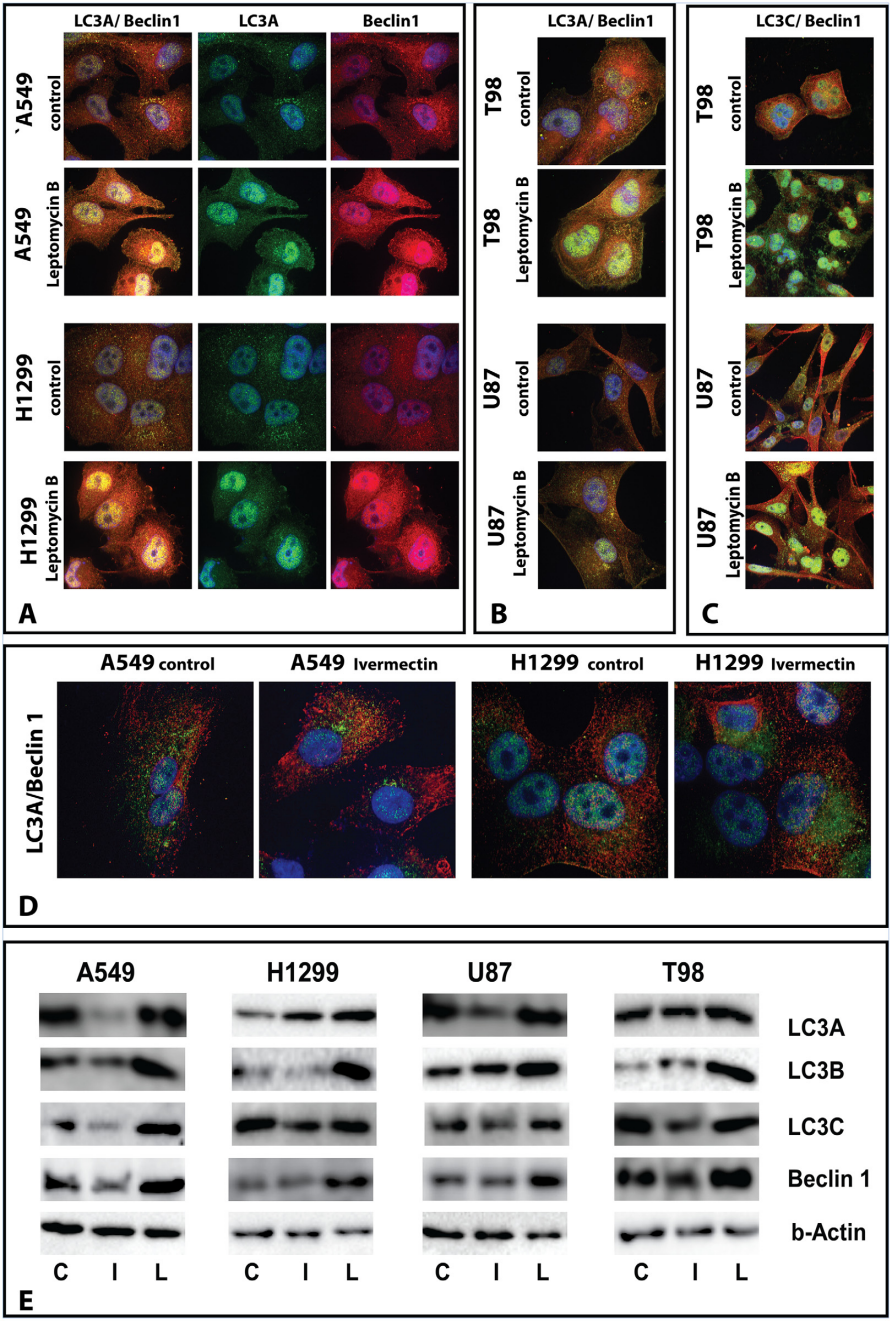
**(A,B,C)** 24h incubation with Leptomycin B at 10 ng/ml, results in nuclear accumulation of LC3A, LC3C and Beclin-1 in lung A549,H1299 and glioblastom U87,T98 cell lines. (D) 24h incubation with Ivermectin at 100μg/ml, reduced the nuclear expression of LC3A/Beclin-1 in A549 and H1299 cell lines and increased the perinuclear accumulation of LC3A in the H1299 cell line. **(E)** Western blots of the nuclear cell fraction confirm accumulation of LC3s and Beclin-1 in lung and glioblastoma cell lines after incubation with Leptomycin B. The reduction of proteins after incubation with Ivermectin was not consistent in all cell lines.

LC3B was poorly expressed in the nuclei but strongly expressed in the nucleolar regions, an area that was negative for LC3A. Nucleolar strong staining is evident in 60–95% of cell depending on the cell line. For example the A549 lung cancer cell line exhibits this staining pattern in 60% of cultured cells, whilst this is as high as 95% in the lung cancer H1299 cell line. Fig 2B1–2B6 show a typical pattern of expression in 4-colour confocal microscopy. Ki67 stains nucleoli and colocalizes with LC3B, but not LC3A. Treatment of cells with Actinomycin D (Fig 2C1) that damages nucleoli resulted in lack of LC3B and Ki67 localization in the nuclei. In contrast, exposure of cells to acidic conditions (pH = 6.5) or hyperthermia (40°C) for 24h did not abrogate the distribution of LC3B in the nucleoli or of LC3A in the nuclei (Fig 2C2 and 2C3). Western blot analysis in the nuclear fraction confirmed the presence of the LC3B protein (Fig 3).

### Expression of LC3C

**Fig 4:**
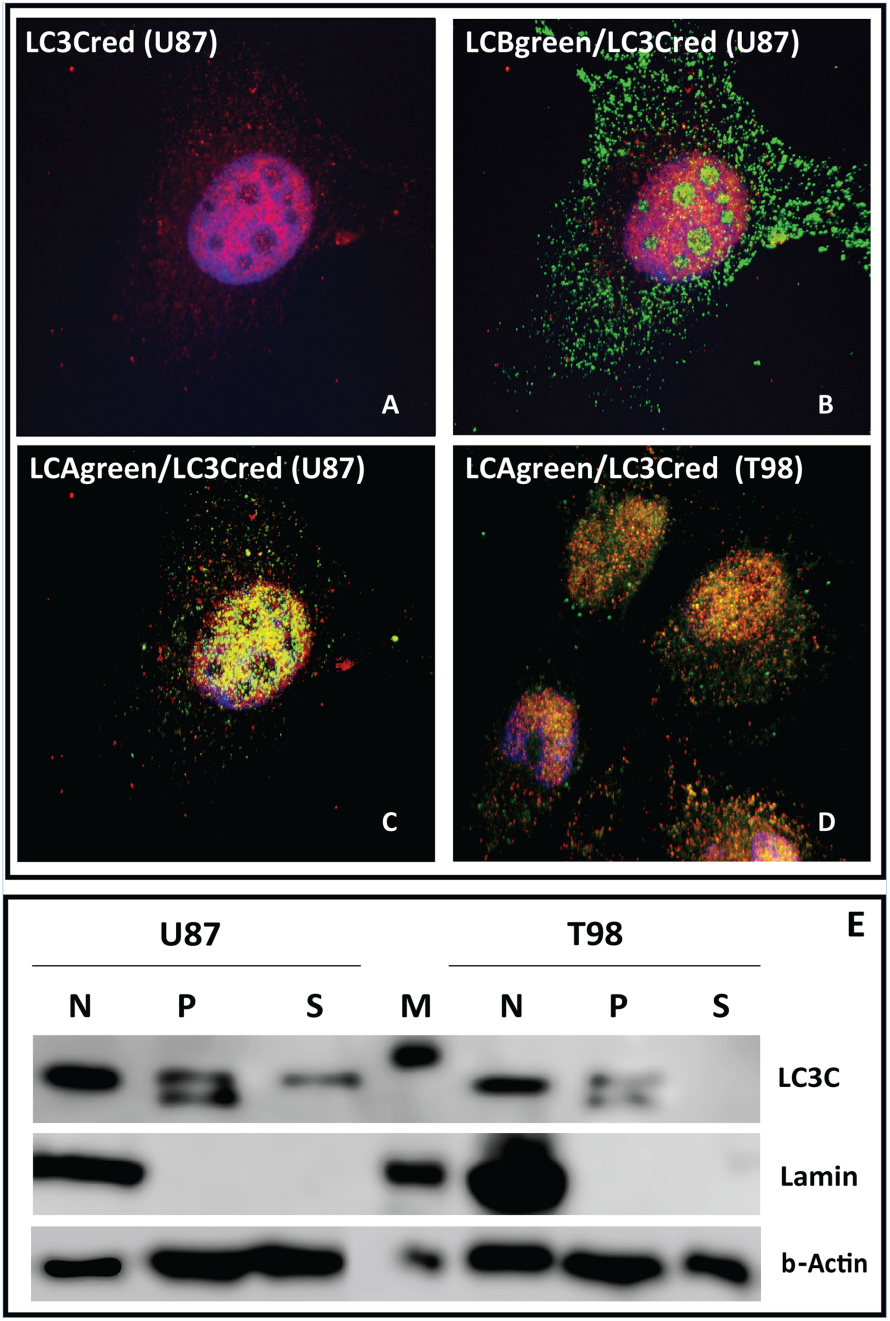
**(A)** Cytoplasmic and nuclear staining of LC3C (red) in U87 cell line. **(B)** Double LC3C (red) and LC3B (green) immunostaining in U87 cells showing lack of co-localization between the two proteins, both in the cytoplasm and the nuclear/nucleolar regions. **(C,D)** Double LC3C (red) and LC3A (green) immunostaining showing co-localization between the two proteins, mainly in the nuclear area (U87 and T98 cell lines). **(E)** Western blot for LC3C in the nuclear (N), Pellet (P) and Soluble (S) fraction of U87 and T98 cells. ‘M’ is the marker and Lamin is used as a control to confirm the presence of nuclei only in the nuclear fraction.

LC3C was clearly expressed in the cytoplasm and more intensively in the nuclei of cells (Fig 4A). Western blot analysis showed a clear presence of LC3C in the nuclear fraction (Fig 4E). There was no evident localization in the nucleolar areas and in double immunofluorescence with LC3B there clearly lack of collocalizaton in the nucleoli and in the cytoplasm (Fig 4B). In double immunofluorescence, LC3C was extensively co-localized with the LC3A in the nuclear area (Fig 4C and 4D), while their colocalization was minimal in the cytoplasm. The prominent expression of LC3C in the nuclei was also confirmed in western blot analysis (Fig 4E). These findings were confirmed in all cell lines examined. Table 1 summarizes the distinct expression patterns for LC3A, LC3B and LC3C.

**Table 1:** Prevalent subcellular patterns of LC3 expression.

	Cytoplasmic		Nuclear	
	Homogeneous	Perinuclear	Nuclear	Nucleolar
**LC3A**		+	+	
**LC3B**	+			+
**LC3C**	+		+	

### LC3 nucleo-cytosolic trafficking

Cells were incubated for 24h with Leptomycin B, a specifically and potent inhibitor of the CRM1/exportin 1 pathway of nuclear export. At 10 ng/ml for 24h incubation, accumulation of LC3A in the nuclei was increased, suggesting a blockage of LC3A extrusion kinetics from the nuclei (Fig 3A). Of interest, LC3A nuclear accumulation showed an intense co-localization with the autophagy signaling protein Beclin-1, and similar kinetics under Leptomycin B exposure (Fig 3B). Similar kinetics and colocalization patterns with Beclin-1 was confirmed for the LC3C protein (Fig 3C).

Ivermectin, on the other hand, is a potent inhibitor of importin *α*/*β* (Imp*α*/*β*1) nuclear import dependent transport. Cells were incubated for 24h with Ivermectin at various concentrations. Following a 24h incubation at 100 μg/ml, the accumulation of LC3s and of Beclin-1 in the nuclei was decreased, while cytoplasmic staining was increased (Fig 3D and 3E). The findings, however, varied among cell lines, as decrease expression was not confirmed for all proteins in all cell lines.

## Discussion

In humans the LC3 gene family has five members, the LC3Av1 (variant 1; NM_032514), LC3Av2 (variant 2; NM_81509), LC3B (NM_022818), LC3B2 (NM_001085481) and the LC3C (NM_001004343). The geneloc for LC3A is 20.q11.22 whereas for the MAP1LC3B is 16q24.2 and MAP1LC3C is 1q43. Both LC3A and LC3B are differentially expressed in normal tissues [3,4,7,8]. Further, the LC3C (the third isoform of the LC3 family) is believed to be poorly or not expressed in most normal tissues [3,4,7]. However, only the LC3A (variant 1), LC3B and LC3C has been show to have post translation modification and generate the form-II [4]. Further, going back to the literature [3,7,8] the post translation site for the MAP1LC3B only is not conserved. For example He et al. 2003 [3] reported that essential site for the distinct post-translation modification of MAP1LC3B is Lys-122 rather than the conserved Gly-120, which reported for the MAP1LC3A and MAP1LC3C. On the other hand Tanida et al. 2005 [8] reported that the carboxyl terminus of MAP1LC3B is cleaved to expose Gly(120) for further ubiquitylation-like reactions. In these two publications it seams that the MAP1LC3B produce the LC3B-II, while the post-translational modification site is not conserved.

In the literature the best-studied endogenous autophagic marker is LC3B. It is stressed, however, that the majority of the studies performed use anti-LC3 antibodies presumed to be anti-LC3B and not anti-LC3A, without having validated their specificity [9,10,11]. There is a high identity between the LC3A and LC3B isoform, indicating that the epitopes used to develop specific antibodies are crucial. According to our results some antibodies are not specific, recognizing both isoforms with a varying sensitivity, while others are able to detect the specific isoforms of LC3A and LC3B. In any case, specifying the LC3-type studied would be unnecessary if indeed the different LC3 proteins had the same expression pattern and biology in normal and cancer tissues. However, this is not the case as shown in the current study.

In a previous study of ours [12], examining the autophagic flux in mouse tissues after various stresses, immunoblotting work showed that LC3A protein follows discrete patterns of LC3A-I and LC3A-II changes in liver, lung, kidney and heart tissues of mice. This stressed that LC3A is also stress inducible and has specific patterns of expression of its soluble and autophagosome bound form. Herein, in confocal microscopy using validated specific anti-LC3 antibodies we confirmed that LC3A and LC3B autophagosomes are distinct, never composed by both proteins and have distinct subcellular distribution. The LC3A prevalent perinuclear expression contrasts with the rather homogeneous distribution of LC3B throughout the cytoplasm, suggesting a distinct biological role between the two autophagosome types. Of interest, in a paper by Bai et al. 2012 [4]. LC3A and LC3B frequently co-localized in the same puncta in starvation conditions (67%) while in basal conditions the co-localization was at 13% in Saos-2 cells. We also noted, in confocal microscopy, a sporadic colocalization of LC3A and LC3B, giving the impression of a large LC3B+ autophagosome digesting an LC3A+ one.

The cytoplasmic biology of LC3 mediated autophagy is well studied. The role of LC3s in the nuclei remains obscure. Karim et al first detected the LC3-II protein form in the nuclei of rat hepatocytes [13], which is in accordance with our unpublished experience with BALB/c mouse hepatocytes. Drake et al reported that although LC3A and B share no known nuclear localization signal, a nuclear export signal may exist, located at residues 63 to 73 of human LC3 [14]. EGFP-LC3 protein was clearly localized in the nuclei of COS-7 cells. In the current study, using a wide range of cancer cell lines, as well as normal human fibroblasts and endothelial cells, we confirmed the nuclear presence of all three LC3 proteins. There was, however, a striking differential localization of the LC3B protein that preferentially localized in the nucleolar regions, while the A and C forms were localized in the extra-nucleolar nuclear area. The role of this LC3B nucleolar expression remains a mystery, just like the role of LC3A and LC3C in the rest of nucleus. He et al proposed that nuclear LC3 may be involved in the control of cell proliferation in promyelocytic leukaemia [15].

We investigated whether known nuclear transport pathways are involved in the nucleo-cytosolic trafficking of LC3s. The nuclear protein import and export pathway mediated by nuclear pore complexes (NPC) was studied, using the agent Leptomycin B, an antifungal antibiotic, that specifically and potently inhibits the CRM1/exportin 1 pathway of nuclear export by directly binding the CRM1 protein [16,17]. Following 24h incubation of lung and glioblastoma cell lines, a substantial accumulation of LC3A, LC3C and of Beclin-1 proteins was evident by confocal microscopy, which was also confirmed on western blot analysis. This contrasts with the finding by Drake et al [14], where a short 3h incubation of COS-7 and HeLa cells with Leptomycin did not result in EGFP-LC3 nuclear accumulation. Beclin-1 contains a leucine-rich nuclear export signal and disruption of CRM1 function results in nuclear accumulation of Beclin-1 [18]. Of interest, Beclin-1 co-localizes in the nuclei with LC3A and LC3C, while no prominent co-localization was noted in the cytoplasm. Whether Beclin-1 binding to LC3 is required for nuclear entrance of a complex demands further investigation.

Ivermectin, on the other hand, is a potent inhibitor of importin *α*/*β* (Imp*α*/*β*1) nuclear import dependent transport, with no effect on proteins containing nuclear localization signal (NLSs) recognized by alternative nuclear import pathways [19] Incubation of cancer cell lines with Ivermectin resulted in decreased expression of LC3s and Beclin-1 in the nuclei, a result however that varied among cell lines and proteins examined.

Since endogenous LC3 proteins are major markers of autophagy, it is essential to check the specificity of the antibodies used when performing experiments for LC3A or LC3B processing in response on different type of stressors. These molecules are not identical and may have distinct biological roles, not well clarified as yet. The nucleolar LC3B localization and the nuclear LC3A, LC3C and Beclin-1 accumulation found in the current study provide a basis for further studies on the distinct biological role these proteins may have in normal and cancer tissues.

## Materials and Methods

### Cell line cultures

Lung cancer cell lines A549 (human lung adenocarcinoma, CLS GmbH, Germany), and H1299 (human non-small cell lung carcinoma, ATCC), glioblastoma cell lines U87MG (human glioblastoma-astrocytoma, CLS GmbH, Germany) and T98G (human glioblastoma multiforme, ATCC), breast cancer cell lines (HER2+ BT474, HER negative MDA-MB-231, DA3; ATCC), as well as embryonic MRC5 fibroblasts cell lines (CLS Cell Line Service, Germany) were cultured using DMEM basal medium (31885–023, Gibco). HUVEC cells (CLS Cell Line Service, Germany) cultured in specific medium (EBM™-2 Basal Medium with EGM™-2 SingleQuots™ of Growth Factors; Lonza) were also studied. The basal culture medium was supplemented with 10% FBS (FB-1000/500, Biosera), 100 units/ml Penicillin and 100 μg/ml Streptomycin (15140–122, Gibco) and 2 mM L-Glutamine (25030, Gibco). Cells were maintained at standard conditions, 37°C, 5% CO2 in humidified atmosphere and were used upon reaching 70–90% confluence.

### Chemicals

The cell cultures were treated separately, as specified for 24h incubation time with Leptomycin B (L2913, Sigma-Aldrich) at a final concentration of 20 ng/ml and Ivermectin (I8898, Sigma-Aldrich) at a final concentration of 100 μg/ml.

### Identification of LC3A, B and C specific antibodies

To test specificity of commercially available antibodies regarding their specificity for LC3A or LC3B the following antibodies were used against the commercial available human recombinant proteins LC3A (H00084557-P01, Abnova) and LC3B (H00081631-P01, Abnova):

1. The rabbit polyclonal to LC3A (1:30.000, ab62720, Abcam, a synthetic peptide PSDRPFKQRRSFADR conjugated to KLH by a Cysteine residue linker, corresponding to amino acids 2–15 of Human MAP1LC3A)
2. The rabbit polyclonal to LC3A (1:2500, 1805a, Abgent, a synthetic between 97~120 amino acids from the C-terminal cleavage site of human cleaved-LC3A use as an epitope)
3. The rabbit monoclonal to LC3A (1:1000, ab52628, Abcam)
4. The rabbit polyclonal to LC3B (1:5.000, NB600-1384, Novus, a synthetic peptide made to the N-terminal region of the human LC3, isoform B protein),
5. The rabbit polyclonal to LC3B (1:5.000, L7543 Sigma-Aldrich, a synthetic peptide corresponding to amino acids 2–15 of human LC3B, conjugated to KLH)
6. The rabbit polyclonal to LC3B (1:5000, NB100-2220, Novus, a synthetic peptide made to an N-terminal portion of the human LC3B protein sequence between residues 1–100)
7. The rabbit polyclonal to LC3 (1:5.000, L8918, Sigma-Aldrich, a synthetic peptide corresponding to amino acids 36–49 of human LC3A isoform a, conjugated to KLH via C-terminal cysteine residue).
8. The mouse monoclonal antibody against to the LC3B (1:5000, 5F10, Nanotools, a synthetic peptide made to an N-terminal portion of the human LC3B protein).

Regarding the anti-LC3C anibody, we used the rabbit polyclonal (18726-1-AP, Proteintech Europe) antibody. The specificity of the anti-LC3C antibody used has been previously reported (http://www.ptglab.com/Products/Search.aspx?key=LC3C).

### Exposure to Bafilomycin

LC3A and LC3B were analyzed in cell lines under exposure to the autophagy disrupting agent bafilomycin A [8]. Bafilomycin A is a specific inhibitor of the vacuolar type H(+)-ATPase (V-ATPase) in cells, inhibits the acidification of organelles containing this enzyme (such as lysosomes and endosomes) and, furthermore, inhibits the fusion of autophagososomes with the lysosomes [8]. Assessment was performed 24 hours after incubation of cells with 100 nM bafilomycin.

### SDS-PAGE and Immunoblotting

Cells were washed with PBS twice and lysed in a sucrose-based lysis buffer (0.25 M sucrose, 25 mM Tris-HCl, pH 7.4) containing protease inhibitors (complete mini protease inhibitor cocktail, Roche Diagnostics GmbH) and phosphatase inhibitors (phosphatase inhibitor cocktail, Cell Signaling Technology). A differential centrifugation of the whole-cell lysates led to nuclear, supernatant (cytoplasmic-water soluble proteins) and pellet (membrane proteins) fractions. Protein quantification was performed according to the Pierce™ BCA Protein Assay Kit (#23225, Thermo Scientific).

Protein samples were separated on discontinuous SDS gels using 10% separating gel for Beclin-1 while for LC3A, LC3B and LC3C 12.5% separating gel was used. Moreover, 5% stacking gel was used. Forty nanograms of samples analyzed on the gel. Immunoblotting was performed utilizing PVDF-PSQ membranes (Millipore Corp.). Then, membranes were blocked with 5% non-fat dry milk in 150 mM NaCl, 10 mM Tris, pH 7.5 (TBS) and 0.1% (v/v) Tween-20 at room temperature for 2 hours followed by the hybridization overnight at 4°C with primary antibodies. The membranes were then hybridized for 2hr at room temperature with the secondary antibody, goat polyclonal to rabbit IgG-HRP (1:3.000, Biorad, 1706515, USA) or goat polyclonal to mouse IgG-HRP (1:3.000, Biorad, 1706516, USA). Bands were developed using Chemidoc MP Imaging System (Biorad, USA).

The primary antibodies used were: i) rabbit polyclonal to LC3A (1:1.000, ab62720, Abcam, a synthetic peptide PSDRPFKQRRSFADR conjugated to KLH by a Cysteine residue linker, corresponding to amino acids 2–15 of Human MAP1LC3A), ii) mouse monoclonal antibody to LC3B (1:1.000; LC3B 5F10 Nanotools), iii) rabbit polyclonal antibodies to MAP1LC3C (1:1.000, ProteinTechLab) and iv) rabbit polyclonal antibody to Beclin-1 (1:2.000, A303-673A, Bethyl Laboratories, Inc., USA)

Each of these blots was then stripped, dried overnight, re-hybridized with mouse monoclonal antibody to Actin beta (1:5000, NB600-501, Novus Biologicals).

### siRNA

LC3A siRNAs were pooled as (5′-GCGAGUUGGUCAAGAUCAUTT-3′), (5′- GCUUCCUCUAUAUGGUCUATT-3′), (5′-CCUGCUGUGUGGUUCAUCUTT- 3′), (5′-GCUGUAAGGAGGUACAGCATT-3′), and LC3B siRNA were respectively pooled as (5′-GCCCUCUACUGAUUGUUAATT-3′), (5′-CUCCCUAAGAGGAU CUUUATT-3′), (5′- GCCUGUGUUGUUACGGAAATT- 3′). These were custom synthesized from Shanghai GenePharma Co., Ltd (China). These were used at 20–50 nM to transfect cells using HiPerfect (QIAGEN) for 24 h, while the silencing efficiency of siRNAs was confirmed both by confocal microscopy and western blot after 24 h.

### Confocal immunofluorescence and Image analysis

For immunofluorescence staining, cells were grown on No. 1.5 glass coverslips, fixed in 3.7% paraformaldehyde/PBS pH 7.4 for 20 min at 37°C and then permeabilized in PBS/0.1% v/v Triton X-100 pH 7.4 for 5 min at room temperature. In addition, cells were blocked in PBS/5% w/v BSA pH 7.4 for 20 min and stained with various primary antibodies: anti-ki67 (MIB1) mouse monoclonal (1:150; DAKO), anti-LC3A rabbit polyclonal (1:500; Abcam), anti-LC3C rabbit polyclonal (1:200; Proteintech Europe), anti-LC3B mouse monoclonal (1:200; Nanotools) and anti-Beclin-1 rabbit polyclonal (1:100, ab62557, Abcam) for 1 h at room temperature.

Cells were washed in PBS pH 7.4, incubated with appropriate CF 488 and 564 secondary antibodies at RT and DNA was counterstained with Hoechst 33342 (1 μg/ml; Sigma-Aldrich). After final washes coverslips were mounted in homemade Mowiol mounting medium. Imaging was performed on a customized Andor Revolution Spinning Disk Confocal System built around a stand (IX81; Olympus) with a 60x lens and a digital camera (Andor Ixon+885) (CIBIT Facility, MBG-DUTH). Image acquisition was performed in Andor IQ 2 software. Optical sections were recorded every 0.3 μm. All confocal microscopy images presented in this work are 2D maximum intensity projections of z-stack images, and image analysis for the obtained data sets has been performed using ImageJ 1.47v (National Institute of Health, USA). Co-localisation analysis images and calculation of the Pearson’s coefficient for the analyzed images were performed using a combination of the Colocalization Finder and the Coloc 2 plugins in ImageJ.

### Ethics

The study has been approved by the Democritus University of Thrace Research Ethics Committee.

## References

[1] Klionsky DJ, Emr SD. Autophagy as a regulated pathway of cellular degradation. Science 2000, 290:1717–1721. 11099404 10.1126/science.290.5497.1717 PMC2732363

[2] de Duve C, Pressman BC, Gianetto R, Wattiaux R, Appelmans F. Tissue fractionation studies. 6. Intracellular distribution patterns of enzymes in rat-liver tissue. Biochem J 1955; 60: 604–617. 13249955 10.1042/bj0600604 PMC1216159

[3] He H, Dang Y, Dai F, Guo Z, Wu J, She X, et al Post-translational modifications of three members of the human MAP1LC3 family and detection of a novel type of modification for MAP1LC3B. J Biol Chem 2003; 278: 29278–29287. 12740394 10.1074/jbc.M303800200

[4] Bai H, Inoue J, Kawano T, Inazawa J. A transcriptional variant of the LC3A gene is involved in autophagy and frequently inactivated in human cancers. Oncogene 2012; 31:4397–408. 10.1038/onc.2011.613 22249245

[5] Ichimura Y, Kirisako T, Takao T, Satomi Y, Shimonishi Y, Ishihara N, et al A ubiquitin-like system mediates protein lipidation. Nature 2000; 408:488–492. 11100732 10.1038/35044114

[6] Kabeya Y, Mizushima N, Ueno T, Yamamoto A, Kirisako T, Noda T, et al LC3, a mammalian homologue of yeast Apg8p, is localized in autophagosome membranes after processing. EMBO J 2000; 19:5720–5728. 11060023 10.1093/emboj/19.21.5720 PMC305793

[7] Wu J, Dang Y, Su W, Liu C, Ma H, Shan Y et al Molecular cloning and characterization of rat LC3A and LC3B—two novel markers of autophagosome. Biochem Biophys Res Commun 2006; 339: 437–442. 16300744 10.1016/j.bbrc.2005.10.211

[8] Tanida I, Ueno T, Kominami E. Human light chain 3/MAP1LC3B is cleaved at its carboxyl-terminal Met121 to expose Gly120 for lipidation and targeting to autophagosomal membranes. J Biol Chem 2004; 279: 47704–47710. 15355958 10.1074/jbc.M407016200

[9] Ferguson CJ, Lenk GM, Meisler MH. Defective autophagy in neurons and astrocytes from mice deficient in PI(3,5)P2. Hum Mol Genet 2009; 18: 4868–4878. 10.1093/hmg/ddp460 19793721 PMC2778378

[10] Aoki H, Kondo Y, Aldape K, Yamamoto A, Iwado E, Yokoyama T et al Monitoring autophagy in glioblastoma with antibody against isoform B of human microtubule-associated protein 1 light chain 3. Autophagy 2008; 4: 467–475. 18259115 10.4161/auto.5668

[11] Barth S, Glick D, Macleod KF. Autophagy: assays and artifacts. J Pathol 2010; 221: 117–124. 10.1002/path.2694 20225337 PMC2989884

[12] Zois CE, Giatromanolaki A, Sivridis E, Papaiakovou M, Kainulainen H, Koukourakis MI. Autophagy flux in normal mouse tissues: focus on endogenous LC3A processing. Autophagy 2011; 7: 1371–1378. 10.4161/auto.7.11.16664 21997374

[13] Karim MR, Kanazawa T, Daigaku Y, Fujimura S, Miotto G, Kadowaki M. Cytosolic LC3 ratio as a sensitive index of macroautophagy in isolated rat hepatocytes and H4-II-E cells. Autophagy 2007; 3:553–560. 17617739 10.4161/auto.4615

[14] Drake KR, Kang M, Kenworthy AK. Nucleocytoplasmic distribution and dynamics of the autophagosome marker EGFP-LC3. PLoS One 2010;5:e9806 10.1371/journal.pone.0009806 20352102 PMC2843706

[15] He W, Hu CX, Hou JK, Fan L, Xu YW, Liu MH et al Microtubule-associated protein 1 light chain 3 interacts with and contributes to growth inhibiting effect of PML. PLoS One 2014;9:e113089 10.1371/journal.pone.0113089 25419843 PMC4242537

[16] Stade K., Ford CS, Guthrie C, Weis K. Exportin 1 (Crm1p) is an essential nuclear export factor. Cell 1997;90:1041–1050 9323132 10.1016/s0092-8674(00)80370-0

[17] Kudo N, Matsumori N, Taoka H, Fujiwara D, Schreiner EP, Wolff B et al Leptomycin B inactivates CRM1/exportin 1 by covalent modification at a cysteine residue in the central conserved region. Proc Natl Acad Sci U S A 1999;96:9112–9117. 10430904 10.1073/pnas.96.16.9112 PMC17741

[18] Liang XH, Yu J, Brown K, Levine B. Beclin-1 contains a leucine-rich nuclear export signal that is required for its autophagy and tumor suppressor function. Cancer Res 2001;61:3443–3449. 11309306

[19] Wagstaff KM, Sivakumaran H, Heaton SM, Harrich D, Jans DA. Ivermectin is a specific inhibitor of importin α/β-mediated nuclear import able to inhibit replication of HIV-1 and dengue virus. Biochem J 2012; 443:851–856. 10.1042/BJ20120150 22417684 PMC3327999

